# The virtuoso art of bricolage research

**DOI:** 10.3389/fpsyg.2022.1068703

**Published:** 2022-11-17

**Authors:** Smadar Ben-Asher

**Affiliations:** The School of Advanced Studies, Achva Academic College, Arugot, Israel

**Keywords:** bricolage research, multidirectional thinking, holistic research, involved researcher, wild thinking

## Introduction

Originally, the term “bricolage” referred to a variety of non-professional occupations carried out in an improvised and amateurish way. It has been used to describe a postmodernist technique of creatively recycling leftover items. The technique can be applied in a variety of fields, including visual art, industrial design, music, architecture, philosophy, and linguistics.

The term was coined in the field of social sciences by Claude Lévi-Strauss (1908–2009), the founder of the structuralist school, considered to be the leading anthropologist of the 20th century. In 1962, Lévi-Strauss presented his concept of “wild thinking” (Levi-Strauss, 1962), based on his travel diary, *Tristes Tropiques* (Lévi-Strauss, 1955), which he wrote in the rainforests of Brazil. In the diary, Lévi-Strauss related his experiences combining deep philosophical thought, prose, and anthropological investigation, all serving as a tool for describing the phenomena he observed. In his opinion, it is precisely the wild thinking, which is not based on ready-made patterns, that allowed him, as a researcher, to explain the world to himself and integrate into it. Lévi-Strauss referred to researchers who present only facts and data as “engineers;” by contrast, to those who make use of different materials as craftsmen or *bricoleurs*. Lévi-Strauss contrasted the concept of the “scientific mind” with “wild thinking.” In his opinion, scientists' resort to previous systems of theoretical and practical knowledge when they lack the information needed to complete a picture. The phrase wild thinking legitimizes the researcher's right to freedom of action, without rigid boundaries.

Both the engineer and the bricoleur live in a limited reality because every choice of tools and materials, whether facts or history, is personal and therefore not absolute or universal. Researchers using the bricolage methodology dare to take materials from their immediate environment, “whatever they can lay their hands on,” even if these are vastly different, to assemble them and create a new innovative whole. Lévi-Strauss described a “significant bricoleur” as one who is skilled in performing a large number of varied tasks. Unlike the engineer, this researcher is not subject to the availability of raw materials and tools prepared in advance for the project (Levi-Strauss, 1962). Lévi-Strauss described the bricolage methodology as a combination of the researchers' imagination with all the tools of knowledge at their disposal, using a rich repertoire of rituals, meaningful objects, observations, and social practices. To all these are added structured conversations and spontaneous interviews, institutional knowledge, and informal knowledge (Rogers, 2012; Lévi-Strauss, 2014). The concept of savage thinking caused animated disputes between philosophers and social scientists and was challenged by the range of possibilities for observing the world that Lévi-Strauss presented and believed in Lévi-Strauss (1983, 2014).

Support for Lévi-Strauss's view was received in the early 1970s by the philosopher of science Feyerabend (1974) in his book Against Method. Feyerabend claimed that science is a free creation of the human spirit and should not be limited by the general do's and don'ts of scientific research methods. In his opinion, it is not possible to separate fact from theory because there is nothing we know for sure. Feyerabend's starting point is ethical: it is not moral to limit the creativity of human beings. According to him, being a scientist is closely related to the ability of being moral, which requires complete openness and the absence of physical or intellectual coercion.

The research concept proposed by Lévi-Strauss found support in the work of Clifford James Geertz (1926–2006), one of the founders and leaders of symbolic anthropology. Geertz claimed that culture is transmitted in a system of concepts and symbolic forms that suit each society differently. Reality is full of symbols, therefore only a thick description of reality can lead to its understanding (Geertz, 1980). The wide range of interpretive practices that Geertz proposed is largely similar to the bricolage worldview described by Lévi-Strauss. Geertz called for a multi-directional scientific investigation that combines the methodologies of quantitative sciences with qualitative research. According to Geertz, there is no single absolute and correct reality, but different interpretations of reality must be explored through the construction of an experiential memory that will help describe and explain things with their many facets and all their complexity. Geertz (1994) claimed that the idea of many structures of personal and cultural reality is at the basis of the qualitative concept. The multiplicity of points of view demands a holistic approach, therefore it is not possible to expropriate individual variables from the overall context. To understand significant moments in a person's life, it is necessary to reconstruct one's personal experience and reflective understandings, and examine them in the context of cultural and historical texts and products or those conveyed by figurative structures of objects, buildings, monuments, or paintings. The starting point of qualitative research, to which the bricolage method belongs, is a naturalistic interpretive perspective, according to which the investigation is carried out within the natural world with as little intervention as possible.

The anthropological approach of Lévi-Strauss and Gratz also finds deep roots in the epistemological concepts of Aristotle and Plato, in the fourth century BC. Epistemology concerned the nature of knowledge, its sources, limits, and reliability (Steup, 2012). Preoccupation with the epistemologies of the elements of knowledge resulted in many studies that dealt with an in-depth examination of the four elements of knowledge: its certainty, complexity, source, and justification (Hofer, 2002).

Sabar Ben-Yehoshua (2016) described the use of the qualitative research approach as part of the research complex that includes a combination of methods, when there is agreement that each practice allows observation and examination from different angles. Bricolage research encourages the researcher not to be bound by orthodox and rigid approaches, which are at times external to the need and context being studied but to use what is available and thus deepen the research (Berry, 2004).

Denzin and Lincoln (1998, 2011) identified five areas of research writing in which the characteristics of bricolage can be found: theory, methodology, interpretation, political positions, and narratives. Adopting the bricolage approach means agreeing with the assumption that every study is subjective because it is influenced by the researcher's personal history, biography, gender, social class, race and ethnicity, as well as by the people in the society being studied (Denzin and Lincoln, 2011).

A bricolage approach requires a deep understanding that there is no one correct description of an event. The researcher's point of view shifts between the theoretical infrastructure and the observation of the phenomenon, the information that arises in the context of the researched topic, the data analysis, the researcher's point of view, the literary genre that is relevant to different parts of the research, and the language in which it is presented. To this complexity must be added the in-and-out movement, and the positions of the bystander and the participating observer. At times the researcher becomes temporarily the main character of the plot (Spector-Marzel, 2017).

Denzin and Lincoln (2011) reviewed the history of qualitative research and described the bricolage metaphor coined by Lévi-Strauss as having a broad meaning. According to them, the bricolage approach made possible the merging of a large number of disciplines from the humanities and social sciences, as well as multiple methodologies such as ethnography, discourse analysis, deconstruction, folk genealogy, and perspective-changing theories such as feminism, Marxism, and postcolonialism. The boundaries between the social sciences and the humanities became blurred, and researchers from the social sciences began working with the humanities and building semiotic models, theories, and methods of analysis. The bricolage approach to research and writing allows systematic description (*graphy*) alongside personal experience (*auto*) for understanding cultural experiences (*ethno*) (Spector-Marzel, 2017).

Weinstein and Weinstein (1991) argued that the very representation chosen and the interpretation given to the combination of materials is bricolage work because it represents the researcher's impression understanding, and interpretation of the phenomenon under study. The researchers' personal narrative is a significant tool for understanding their point of view when they interpret the events before them. The researchers themselves have an opinion, social position, focus of interest, practices, and methodologies that serve these perceptions.

The bricolage method has encountered difficulties and objections in qualitative research. Pratt, Sonenshein, and Feldman, who have been editors of qualitative journals for many years, in summarizing their professional experience (Pratt et al., 2022), claimed that the reason was probably entirely pragmatic. Qualitative research was based on a wide range of methods (Bansal et al., 2018; Reay et al., 2019), and to “set things straight” and evaluate the studies, the reviewers of the articles required the researchers to use “templates” and “formulas,” renouncing creativity and setting up a research method adapted to the unique conditions of the research and its objective. The most attractive feature of templates is that they simplify research methods. But templates cannot account for the special details and subtle ways that vary from study to study. Pratt et al. (2022) argued that a pattern is at most a map, whereas the study examines an area that is infinitely more complex than the map.

We can conclude that the bricolage approach uses different methodologies that flexibly examine the complex dimensions of a studied phenomenon. The research process is characterized by observation from multiple perspectives that are sometimes competing with each other (Rogers, 2012). The bricolage approach combines in one study many methodological practices and empirical materials, different perspectives, and is best understood as a strategy that adds rigor, breadth, complexity, richness and depth to any investigation (Denzin and Lincoln, 2011).

## Case study: The incident of the naval commandos diving in the polluted Kishon river

To demonstrate the challenges of a complex reality faced by researchers, containing many unplanned constraints and opportunities, we present here a research unit from a broad study conducted between 2008 and 2010 in Israel of a case that caused great public upheaval.

The incident is commonly referred to in Israel as “the Kishon affair.” In May 2000, two Israeli journalists revealed that soldiers had been training for years in a river that was highly contaminated with industrial waste dumped by nearby factories despite the potential danger of contact with polluted waters that could cause serious illnesses, particularly various forms of cancer. The army failed to take action to protect its soldiers (Neumann and Ben-Asher, 2003). Given the highly selective criteria for acceptance into this elite unit, the soldiers had been in excellent health and top physical condition at the time of their recruitment, therefore the high incidence of illnesses and mortality among them could not be explained by random statistics (Ben-Asher and Goren, 2006). Following the journalists' exposé and the soldiers' demand that the state acknowledge its responsibility for the harm, a special commission of inquiry was appointed, headed by former Supreme Court Justice Meir Shamgar. After 3 years of hearings and the report of the Shamgar Commission, the state accepted responsibility for treating the naval commandos who had contracted cancer and for assisting the families of those who subsequently died. The naval commandos' struggle received extensive coverage in the Israeli press for three years, from the publication of the original exposé to the state accepting responsibility (Ben-Asher and Ben-Atar, 2016).

The national press in Israel showed great interest in the fighters who contracted cancer after diving in the Kishon. The press became involved in the case, showed concern, and adopted explicit or implicit positions regarding the ethical aspects of the affair. At the same time, it transpired that the fishermen of the Kishon River who worked in the exact same place where the commandos had trained also suffered from serious illnesses, and many of them died of cancer. Despite the similarity between the fishermen's and the commandos' struggles to force the polluting factories and the state to assume responsibility for harming their health, the fishermen's struggle, which lasted 13 years, did not receive media or public attention. The fishermen's struggle was covered only by the local press, which is distributed locally and is not archived.

The question of the prominence and exclusion of groups in Israel is central in a democratic, liberal country, whose main foundation is the right to equality between citizens. The recognition and recognition of the plight of members of different social groups by members of other groups, and the construction of a reality by the media of a reality in which marginalized groups can be part of the social discourse are worthy goals for building a more just society. In an early study (Ben-Atar and Ben-Asher, 2015; Ben-Asher and Ben-Atar, 2016), we examined whether there is justice and equality among citizens in Israeli society. The study examined whether it is possible that the Israeli media openly and almost explicitly favored one social group (the commandos) over another (the fishermen). We sought to uncover the fishermen's narrative about their struggle and the public attitude toward them, as it is reflected and presented in the national and local media.

## Constructing the research methodology

Constructing a systematic qualitative research methodology should yield a collection of written information from documents, the media, and interviews with the leaders of the group and of the struggle. In the case of the present study, almost every methodical way was blocked:

1. Documents. The documents of the struggle that lasted for years (including the ones submitted to the court) were not systematically collected. One of the old fishermen (at the time of the interview he was over 80 years old and not in good health), who used to be the head of the committee, showed us a disorganized collection of letters and documents. He refused to let us makes copies of them but agreed for us to read them out loud and record the reading. In this way, we were able to create a record of the content of these documents.
2. Legal documents. The attorney who led the fight was a retired judge who voluntarily agreed to represent the fishermen. In a telephone conversation with him, he begged us to come to the district court where every Sunday the hearing of the fishermen's claim was held. The lawyer said that he faced alone 43 lawyers of the petrochemical plants who showed up in court with numerous assistants. Unfortunately, for some time after our conversation, the lawyer was not able to continue the work for health reasons, and a few months later he died. The trial continued with the assistance of a young lawyer who voluntarily assumed the task of representing the fishermen out of ideological and social motives. We were exposed to the legal documents in his possession, with his consent, only about a year after he submitted the summary of the claim to the court.
3. Interviews with the participants of the research, the leaders of the fishermen's struggle. Forty-eight fishermen filed claims in court. In practice, the number of families of the sick or dying from cancer was estimated to be about 60, but some of the families chose not to join the fight after the representatives of the petrochemical plants threatened them with high financial claims if they filed a lawsuit in court. In the absence of a proper organizational structure for the fishermen, and especially for those who were sick or elderly and had already retired from work, we had difficulty reaching and interviewing them. In the past, the older fishermen used to hold a traditional informal meeting on Fridays, which they called “Parliament.” During the period in which we conducted the study, many had already become ill or passed away, and the weekly meetings were discontinued.

Interviews we conducted with several fishermen before our visit to the port revealed that the younger members of the group were organized by a fisherman with a criminal background. He managed the fish trade in a shed where the fishermen assembled, with full control over the product caught by all the fishermen. The same person represented the fishermen in the lawsuit. His interest was strictly financial, without any social orientation, support, or concern for the families of the sick and deceased. We understood that interviews with the fishermen needed his approval and supervision. We were required to work with a person involved in various transactions, most of which were hidden from the state authorities.

4. Appearances in the visual media. Whereas the representatives of the naval commandos were often the guests of television studios, were interviewed, and presented their struggle clearly and eloquently, the representative of the fishermen was never invited to these studios. Few in the Israeli public knew about the fishermen's struggle, which was conducted at the same time as the well-publicized struggle of the naval commandos.
5. Printed press. As noted, the public media refrained from reporting on the fishermen's struggle. The articles published in the local press over the years were not collected in any archive.

We chose to conduct bricolage research in the field, similar to ethnographic research. We sought to examine the struggle of the fishermen with the help of observations, spontaneous authentic interviews and data collection from the field. We did all this using our subjective personal experience and interpretation of it. According to the bricolage methodology, the description must be systematic and include the subjective data of the researcher, the research process, the findings collected and their interpretation, as well as the emotional and cultural experience of the researcher or the critical point of view of the researcher.

## Research process

To demonstrate the bricolage nature of the investigation, we describe one incident that occurred in the course of the research that illustrates the uniqueness of this method.

The researchers were a mother-daughter team, both engaged in the academic field: the mother (the author) is a psychologist who studies the social thinking of marginalized minority groups, especially those affected by illness and bereavement; the daughter specializes in the field of communication. The two researchers were personally and tragically connected with the Kishon affair because the father of the family, who was one of the commandos, died of cancer a few years before the study was conducted and was recognized by the state as an army casualty.

In a roundabout and complex way, we managed to contact the leader of the fishermen and schedule a meeting with him in the morning, in the port at the station where the catch was collected and sent to the markets. The night before we arrived there was a high tide that resulted in a larger than usual quantity of fish. The guard at the entrance of the building where the fish are distributed to the various markets contacted the leader of the fishermen and announced our arrival. He informed us that because of the heavy workload that morning, he would not be able to make time to talk with us, but he agreed to send another fisherman, a man he trusted, for us to interview in his place.

We conducted the interview on a spot covered with grass that separates two roads inside the port. The place was not ideal for holding an in-depth interview. After we introduced ourselves, including our personal connection to the Kishon case, trust appears to have prevailed over suspicion, and the interviewee shared his personal story, including the description of the struggle to recognize the fishermen who were harmed by the polluted Kishon water and fell ill, his father being one of them. Although the interviewee himself was not a key partner in the struggle, he described what he knew about it and the threats that the families of the sick and deceased received from the petrochemical plants to prevent them from joining the legal struggle. At the end of the interview, we asked him if he had come across articles in the local press about the fishermen's struggle. The interviewee confirmed that such articles were collected by the fishermen, although he did not know how many of them have been kept. The interviewee called the leader of the fishermen and after a short conversation asked us to follow him.

He led us to a dark cold store, packed with fresh product, and he climbed up a shady staircase, at the end of the room, to a small office that appeared to be an observation room for the cold store. The leader of the fishermen watched us from this room at the top of the stairs, without coming down. It is reasonable to assume that the appearance of the foreign women caused a measure of concern, which may have dissipated to some degree at the sight of the pregnant belly of the daughter and by the impression we produced on the fisherman who spoke with us earlier, which was likely been conveyed orally to the “observation tower” inside the cold store. A few moments later the messenger fisherman returned holding a black garbage bag: “Take it and return it in half an hour,” he told us.

Outside the dark cold store, we untied the bag and found a treasure in it: a collection of articles published in the local press, photographs, and documents that the fishermen had cut out and kept. It was a unique authentic collection not found anywhere else. On the large table in another office in the port, we spread out all the articles and photographed them with a simple camera, after which we returned the black plastic bag to the guard at the entrance to the fish warehouse.

Unexpectedly and without planning, our research received an empirical basis for a comparative study of the public national and local media in the case of the Kishon fishermen. We examined how the opportunity was used or missed to give a local, weak, and marginalized group a public stage to present its struggle and to win social recognition similar to that of the naval commandos (Ben-Asher and Ben-Atar, 2016).

## Discussion

The present article follows the recommendation of Pratt et al. (2022) to regard the bricolage methodology as an analytical alternative to methodological templates. This approach has the potential to improve the way researchers understand, formulate, and implement methodological choices in their research. Using this approach, they recognize that there are many possible ways of conducting high-quality qualitative research. The metaphor of methodological bricolage provides a framework for thinking about the variety of approaches in ways that instill confidence in the methods used. The bricolage metaphor allows us to refer to researchers as bricoleurs, which focuses attention on their agency, creativity, and craft (Klag and Langley, 2013).

Researchers who adopt the bricolage methodology (for example, Baker and Nelson, 2005; Duymedjian and Rüling, 2010; Pratt et al., 2022) list its three key components: being satisfied with the materials found in the field given the limitations and constraints imposed by reality, utilizing the resources at hand, and combining resources for new purposes. In the short description of the research concerning the part of the media in representing the suffering of the fishermen who came in contact with the polluted water of the Kishon, we can distinguish all three components of bricolage research.

### Constraints

Despite our attempt to meet with the fishermen's leader, the field conditions following the large fish harvest on the day of the meeting prevented the meeting from taking place. The interview with the fisherman who was sent to us in his place was a constraint. For us, the visit to the port was the available resource. Note that in the course of the research, we met with additional sick or old fishermen who had left work, as well as with fishermen who previously belonged to this group but left the work at sea in favor of other jobs.

### Utilization of available resources

Bricolage deals with the available resources and their understanding. The garbage bag in which the articles of the local press about the fishermen's struggle were collected was a resource that could not be obtained by a normal search of public archives, given the absence of such archives of the local newspapers. The articles were cut out and collected by the fishermen unsystematically, and some of them lacked the date of publication, the name of the writer, or even the name of the newspaper in which they were published. Although we cannot be sure that all the articles published in the local press were indeed collected, their large number (32) allowed us to show that whereas the national press failed to express solidarity with a marginalized social group and preferred a sympathetic identification with a group close to the consensus of the national narrative, the local press remained in touch with the common people and expressed sympathy for their struggle. Many articles in the local press presented the fishermen fighting the petrochemical plants as struggling for the principle of justice and not for financial compensation. The articles were important for two reasons: their content and the fact of their publication (Ben-Asher and Ben-Atar, 2016).

### Integration of resources for new purposes

Following the analysis of the articles in the local press, we were also able to identify the weak points in the fishermen's struggle: lack of leadership that can be accepted by the general public as moral and valued, the fishermen's inability to give voice to their struggle in the print and visual media, and the difficulty of a disadvantaged socio-economic group to organize a legal battle against powerful commercial entities. One of the difficulties that stood out following the conversations with the sick fishermen was that of creating a consistent sequence over time of active expression of the struggle. For example, the fishermen were absent from the courtroom during the hearings of their claim for recognition of the harm to their health.

Bricolage involves combining resources for a new purpose. In the bricolage methodology, we creatively incorporated practices designed to fit the research (Locke et al., 2008). For example, when the interviewee who collected some of the documents of the struggle initially refused to let us photograph them, we asked that we read them out loud and record them in this way, to which he consented. It was an analytical move made to identify a problem that arose in the field when we asked to make a record of the documents of the struggle, so that they would be used to substantiate the research claims, but we had to do it in a different way than planned.

Finally, there remains the question of the integrity and reliability of the researchers, their degree of subjectivity, and the part their beliefs, values, and opinions played in the interpretation. The bricolage researcher must display a reflective position, honesty, and transparency. Moving between the individual and the collective, between the inside and the outside should be reflected in the honesty of the researcher. The researcher does not seek to adopt the neutral position of a remote documenter but is a participant, expressing a personal opinion. The involvement of the researchers in the Kishon case and the loss of the father of the family was visible to the research participants and the readers. The personal cost that the Kishon case exacted from the researchers was a source of motivation for research on the topic of state responsibility for the victims of the Kishon water pollution, regardless of class. Writing was also seen as a political act that strives for social change and a social need. The bricolage methodology emphasizes the emotions involved in inquiry (Ellis et al., 2011).

The meanings derived from the understanding of the legitimacy of bricolage research is especially important for researchers at the beginning of their career who prefer to choose “safe” research methods, to use Lévi-Strauss's “engineer” metaphor. Adopting creative and flexible research methods in the spirit of post-positivist approaches attests to the importance of the researcher's freedom. The freedom of unfettered epistemological inquiry is not the exclusive domain of the philosophy of science but can be applied in research in various fields in the social sciences. For example, relational psychotherapy places the one-time relationship between therapist and patient at the center and emphasizes the importance of connections and relationships for creating meaning, contrary previous approaches that advocated neutrality and prevented personal involvement (Mitchell and Aron, 1999). It appears that in several fields simultaneously, the legitimacy of scientific investigation based on an authentic encounter that cannot be planned in advance and cannot be reproduced, yet has the potential to afford new vantage points to the researcher one issues of interest, is gaining strength.

The limitations of methodological research have to do with the great skill required of the researcher, who must navigate with the aid of a roadmap but without marked paths. This demands creativity, navigation skills, reflective ability, and often patience and tolerance for getting lost or wasting time, in the belief that precisely the absence of a well-trodden path may lead to innovation. Additionally, openness is required on the part of the reviewers of the articles to assess them not based on regular patterns known in advance (Pratt et al., 2022).

In conclusion, the theories underlying the bricolage methodology are much more complex than a simple eclectic approach.

Although the concept of bricolage has been used in qualitative research for over 60 years, it is still challenging precisely because of its dynamic nature (Phillimore et al., 2019). This type of research deals with understanding intrapersonal processes, institutional changes and social transformations while combining available research tools and taking advantage of opportunities (Easterby-Smith et al., 2008). The bricolage methodology causes the authors to pay attention to the choice of steps they take, to report transparently on these steps and decisions made “correctly,” and to accept the research as unique, a one-time effort under the given conditions. The researcher is similar to a chess player who chooses the appropriate piece in the unique conditions of the given game, using appropriate strategies to achieve the goal. Similar to the game of chess, although the rules are given, the movement of the pieces is always the result of a combination of analytical and creative thinking.

## Author contributions

The author confirms being the sole contributor of this work and has approved it for publication.

## Conflict of interest

The author declares that the research was conducted in the absence of any commercial or financial relationships that could be construed as a potential conflict of interest.

## Publisher's note

All claims expressed in this article are solely those of the authors and do not necessarily represent those of their affiliated organizations, or those of the publisher, the editors and the reviewers. Any product that may be evaluated in this article, or claim that may be made by its manufacturer, is not guaranteed or endorsed by the publisher.
